# Metabolism in the tumor cell and beyond

**DOI:** 10.1002/1878-0261.13467

**Published:** 2023-06-06

**Authors:** Arkaitz Carracedo

**Affiliations:** ^1^ Center for Cooperative Research in Biosciences (CIC bioGUNE) Basque Research and Technology Alliance (BRTA) Derio Spain; ^2^ CIBERONC Madrid Spain; ^3^ Ikerbasque, Basque Foundation for Science Bilbao Spain; ^4^ Traslational Prostate Cancer Research Lab, CIC bioGUNE‐Basurto Biocruces Bizkaia Health Research Institute Bilbao Spain; ^5^ Biochemistry and Molecular Biology Department University of the Basque Country (UPV/EHU) Bilbao Spain

**Keywords:** anabolism, cancer metabolism, metabolic vulnerability, tumor microenvironment

## Abstract

The process of cellular transformation encompasses the acquisition of key and distinctive features, commonly known as hallmarks of cancer. These hallmarks are supported by tumor‐intrinsic molecular alterations, as well as changes in the microenvironment. Cellular metabolism represents one of the most intimate connections between a cell and the environment. In turn, metabolic adaptation is a research field of increasing interest in cancer biology. In this viewpoint, I will provide a panoramic perspective of the relevance and repercussions of metabolic alterations in tumors with nonexhaustive illustrative examples and I will speculate about the prospects of cancer metabolism research.

## Graphical abstract

Cellular metabolism represents one of the most intimate connections between a cell and the environment. In turn, metabolic adaptation represents a research field of increasing interest in cancer biology. In this viewpoint, a panoramic perspective is provided of the relevance and repercussions of metabolic alterations in tumours with non‐exhaustive illustrative examples and the prospects of cancer metabolism research are discussed.

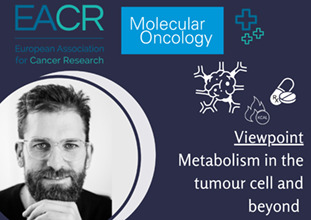

**Abbreviations**
ATP: adenosine TriPhosphate
NAD: nicotinamide adenine dinucleotide
NMDA: *N*‐methyl‐d‐aspartate
ROS: reactive oxygen species

## 1 Cancer cell metabolism and the power to build

When we think of cancer cell metabolism, metabolic programs that enable the production of energy and biomolecules come to mind in the first instance. Otto Warburg reported in 1927 that tumor cells present a propensity toward the anaerobic use of glucose even in the presence of oxygen, a phenotype that is not observed in resting cells and tissues and that represents the beginning of the cancer metabolism field [1]. The intrinsic benefit of this differential use of glucose by cancer cells has been the subject of intense investigation in the past decades, and the data support that the so‐called *Warburg effect* (a) enables rapid ATP production, (b) redirects glucose‐derived carbons for biosynthesis, and (c) reprograms the microenvironment through lactate secretion (see below). The original findings by Otto Warburg are a great example of the unpredictable long‐term impact of *curiosity‐driven research*. The inefficient energetic yield of anaerobic glucose usage is compensated in cancer cells with exacerbated uptake of this nutrient. The development of high‐resolution imaging technologies allows us nowadays to measure the uptake of radiolabelled glucose (or a glucose derivative that is retained intracellularly, 18‐fluoro‐2‐deoxyglucose or FDG) in tissues using positron emission tomography (PET), thus enabling the monitoring of tumor mass and metabolic activity (reviewed in [2]). Inspired by the differential use of glucose by tumor cells, research teams have provided evidence of differential uptake of fatty acids, amino acids, proteins, and other sources of carbon, nitrogen, and energy. In turn, the creative feeding strategies of cancer cells represent an exciting source of tailored biomarkers and therapeutic targets.

Much emphasis has been put on the understanding of the limiting steps for transforming of nutrients into biomass in cancer cells. In this regard, I would highlight three findings in recent years: the production of aspartate in the tricarboxylic acid cycle is necessary for the synthesis of nucleic acids and proteins [3, 4, 5], NAD recycling is required in order to sustain metabolic activity in tumors [6] and the strategy of tumor cells to maximize nitrogen usage and support nucleotide synthesis [7].

## 2 Cancer cell metabolism beyond anabolic programs

Cancer cells rewire their metabolic networks to fuel biomass production. However, metabolic reprogramming has consequences beyond this iconic activity, since not all metabolites regulating cell growth operate by directly producing carbon or nitrogen sources for biomolecules.

**Fig. 1:**
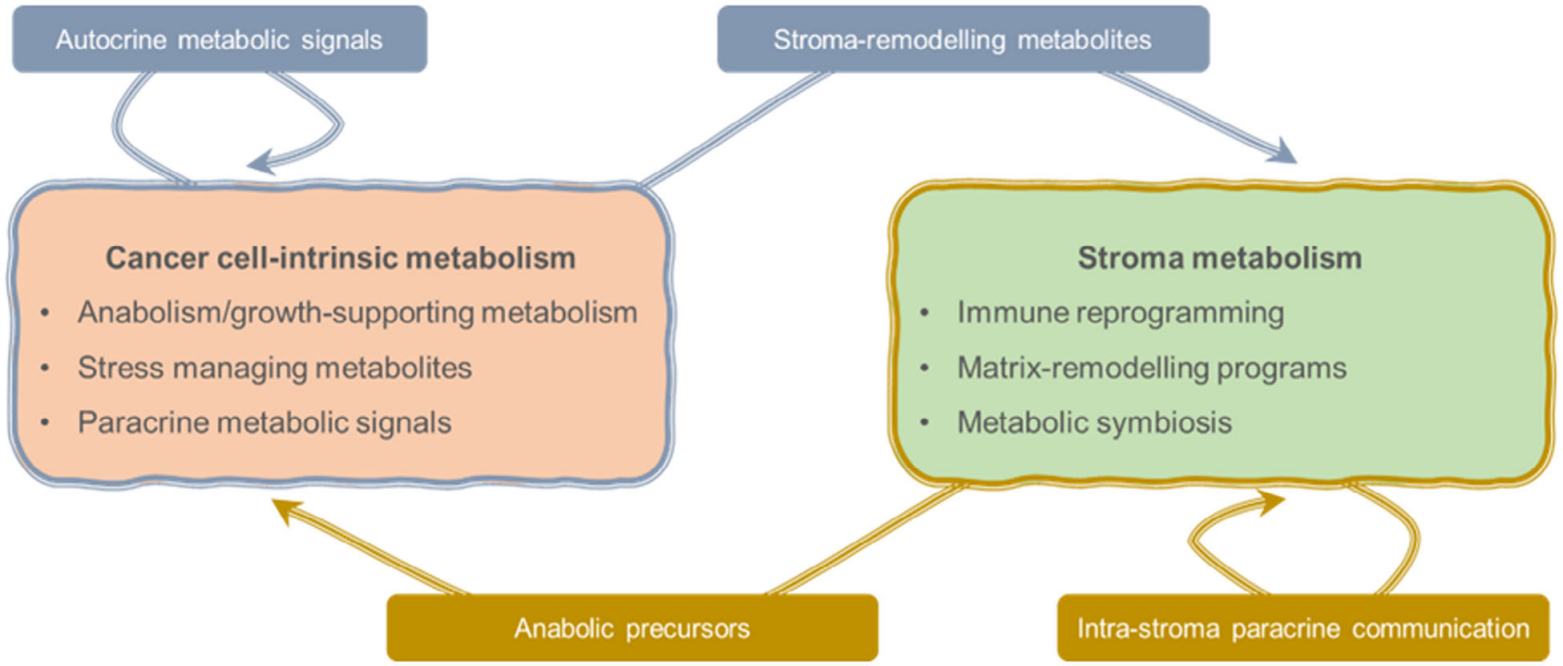
Nonexhaustive illustrative schematic of metabolism‐regulated processes in tumors.

### 2.1 Polyamines

This family of basic polycations is essential for cancer cell proliferation. Although we still lack a full view of their activity, we know that some of their core biological functions emanate from the regulation of transcription and protein translation [8]. These metabolites illustrate the complexity of metabolic programs in the control of cancer cell function beyond the production of biomass. As I will discuss later, these metabolites illustrate the intertwined nature of tumor cell‐intrinsic and extrinsic activities of metabolism.

### 2.2 Antioxidant metabolites

Tumor cells are exposed to exacerbated stress signals when they leave their natural niche, where they struggle for survival. The production of reactive oxygen species (ROS) is a limiting factor for cancer cell survival, a phenomenon that has been found of relevance when tumor cells disseminate through the bloodstream. In this respect, transformed cells that survive in this hostile environment exhibit remarkable metabolic adaptation: i) they decrease mitochondrial activity to reduce ROS production, or ii) activate antioxidant‐producing metabolic programs as a strategy to survive (reviewed in [9]). The paradoxical activity of ROS in cancer, restricting tumor cell survival while increasing the probability of cellular transformation through oxidative damage and mutation burden, has limited the applicability of antioxidants and pro‐oxidants in cancer prevention and therapy. However, the relevance of redox balance in a tumor stage‐dependent manner offers new opportunities for a rational drug combination that includes small molecules targeting this process.

### 2.3 Oncometabolites

The cancer research community has been building the concept of metabolites that enable tumor characteristics. This term is predominantly ascribed to metabolites massively produced by cancer cells harboring specific mutations, such as fumarate (fumarate hydratase‐inactivating mutations) and 2‐hydroxyglutarate (isocitrate dehydrogenase 1/2 neomorphic mutations). However, it is very likely that this term will be refined and accompanied by formal experimental demonstrations that expand the family of oncometabolites to molecules that are aberrantly produced in other cancerous contexts (for a review on oncometabolites [10]).

## 3 Cancer metabolism and the complex dialog with the microenvironment

Beyond the boundaries of the cancer cell plasma membrane, metabolites can exert relevant activities to support crosstalk with the normal cells inhabiting the tumor (the stroma). These metabolic interactions can serve to provide new sources of biomass or to modify the cellular and noncellular microenvironmental landscape. Since noncancerous cells can account for nearly half of the tumor mass, understanding the paracrine crosstalk in cancer can aid in the development of new therapeutic strategies, as demonstrated with the use of immune checkpoint inhibitors. Moreover, the relevance of remodeling the microenvironment in the process of cancer progression leads to the provocative hypothesis that the future of prognostic and predictive biomarkers resides in the use of molecules expressed by the noncancerous cells within the tumor. Some illustrative examples of the metabolic crosstalk between cancer and stroma cells are outlined below.

### 3.1 Metabolic symbiosis

Oxygen and nutrient availability determine the metabolic mode of cancer cells. Within tumors, oxygen tension and nutrient diffusion vary based on vessel density and function, metabolic rewiring, and tissue architecture. This heterogeneity induces a metabolic crosstalk that has been termed metabolic symbiosis. Tumor cells with limited access to oxygen will catabolize glucose and produce lactate, which will be secreted to the extracellular space. Transformed cells in vascularized regions will feed preferentially using the lactate produced by the hypoxic counterparts and oxidize it in the tricarboxylic acid cycle [11]. This metabolic compartmentalization maximizes the use of oxygen and carbons and is not restricted to glucose, since other carbon and nitrogen sources, such as asparagine, exhibit the same compartmentalized behavior in pancreatic cancer [12]. Importantly, metabolic symbiosis in not a dialog limited to cancer cells, but can include stromal cells as a strategy to support tumor growth.

### 3.2 Reprogramming the stromal landscape through metabolic intermediates

Cancer cell‐secreted metabolites can also operate as signaling molecules to alter the function of stromal cells. Three distinct examples are lactate, polyamines, and more recently, *N*‐acetylaspartate. Lactate represents an unanticipated source of carbon for cancer cells, but it also induces the acidification of the extracellular milieu. The paracrine production of lactate exerts pleiotropic activities in stromal cells, from reprogramming macrophages to regulating T‐cell function and fibroblast activation (reviewed in [13]). Similarly, polyamines are growth‐supporting metabolites (see above), but they also serve as paracrine molecules to regulate immune cell function [14]. Lastly, a less studied metabolite, *N*‐acetylaspartate, is produced by the cancer cells at the expense of anabolic intermediates (acetyl CoA and aspartate) and reprograms macrophages to support tumor progression [15]. This is an illustrative set of examples within a larger list of known paracrine‐acting metabolites, a list that is predicted to be extended. Importantly, the secretion of metabolites by cancer cells creates a paradoxical scenario, where tumor cells might sacrifice sources of carbon and nitrogen for anabolism and prioritize the generation of a favorable microenvironmental niche for the disease to flourish.

## 4 The future of cancer metabolism

A nonexhaustive glimpse into the field of cancer metabolism illustrates that metabolites and metabolic enzymes can support the adaptation of cells to an ever‐changing microenvironment (Fig. 1). If we portray tumors as hijackers of pre‐existing molecular programs, we can envision that the study of cancer metabolism can help us identify metabolic processes that are relevant to physiological responses, such as wound healing and tissue regeneration. Conversely, the comprehension of metabolic deregulation in tumors can help identify therapeutic vulnerabilities that can be targeted with metabolic drugs currently used for the treatment of common diseases. Lastly, the influence of systemic metabolism in tumor biology adds complexity to an already complex picture. How do dietary and lifestyle modifications influence cancer responses? How does the tumor alter the metabolism of normal tissues in processes such as cachexia? Can we exploit tailored diets to maximize the effect of anticancer therapies? Some of these questions have begun to receive exciting and innovative answers, but we can expect that curiosity‐driven cancer metabolism research will continue to provide clinically‐relevant evidence that will reformulate our understanding of tumor biology.

## Conflict of interest

The authors declare no conflict of interest.
